# Ultrasound-Assisted Deep Eutectic Solvent Extraction of Phenolic Compounds from Thinned Young Kiwifruits and Their Beneficial Effects

**DOI:** 10.3390/antiox12071475

**Published:** 2023-07-23

**Authors:** Ding-Tao Wu, Wen Deng, Jie Li, Jin-Lei Geng, Yi-Chen Hu, Liang Zou, Yi Liu, Hong-Yan Liu, Ren-You Gan

**Affiliations:** 1 Key Laboratory of Coarse Cereal Processing (Ministry of Agriculture and Rural Affairs), Sichuan Engineering & Technology Research Center of Coarse Cereal Industralization, School of Food and Biological Engineering, Chengdu University, Chengdu 610106, China; 2 Research Center for Plants and Human Health, Institute of Urban Agriculture, Chinese Academy of Agricultural Sciences, National Agricultural Science and Technology Center, Chengdu 610213, China; 3 Singapore Institute of Food and Biotechnology Innovation (SIFBI), Agency for Science, Technology and Research (A*STAR), 31 Biopolis Way, Singapore 138669, Singapore

**Keywords:** unripe kiwifruit, deep eutectic solvent, extraction optimization, polyphenolic, antioxidant activity, anti-inflammatory activity

## Abstract

Fruit thinning is a common practice employed to enhance the quality and yield of kiwifruits during the growing period, and about 30–50% of unripe kiwifruits will be thinned and discarded. In fact, these unripe kiwifruits are rich in nutrients and bioactive compounds. Nevertheless, the applications of thinned young kiwifruits and related bioactive compounds in the food and functional food industry are still limited. Therefore, to promote the potential applications of thinned young kiwifruits as value-added health products, the extraction, characterization, and evaluation of beneficial effects of phenolic compounds from thinned young fruits of red-fleshed *Actinidia chinensis* cv ‘HY’ were examined in the present study. A green and efficient ultrasound-assisted deep eutectic solvent extraction (UADE) method for extracting phenolic compounds from thinned young kiwifruits was established. A maximum yield (105.37 ± 1.2 mg GAE/g DW) of total phenolics extracted from thinned young kiwifruits by UADE was obtained, which was significantly higher than those of conventional organic solvent extraction (CSE, about 14.51 ± 0.26 mg GAE/g DW) and ultrasound-assisted ethanol extraction (UAEE, about 43.85 ± 1.17 mg GAE/g DW). In addition, 29 compounds, e.g., gallic acid, chlorogenic acid, neochlorogenic acid, catechin, epicatechin, procyanidin B1, procyanidin B2, quercetin-3-rhamnoside, and quercetin-3-*O*-glucoside, were identified in the kiwifruit extract by UPLC-MS/MS. Furthermore, the contents of major phenolic compounds in different kiwifruit extracts prepared by conventional organic solvent extraction (EE), ultrasound-assisted ethanol extraction (UEE), and ultrasound-assisted deep eutectic solvent extraction (UDE) were compared by HPLC analysis. Results revealed that the content of major phenolics in UDE (about 15.067 mg/g DW) was significantly higher than that in EE (about 2.218 mg/g DW) and UEE (about 6.122 mg/g DW), suggesting that the UADE method was more efficient for extracting polyphenolics from thinned young kiwifruits. In addition, compared with EE and UEE, UDE exhibited much higher antioxidant and anti-inflammatory effects as well as inhibitory effects against α-glucosidase and pancreatic lipase, which were closely associated with its higher content of phenolic compounds. Collectively, the findings suggest that the UADE method can be applied as an efficient technique for the preparation of bioactive polyphenolics from thinned young kiwifruits, and the thinned young fruits of red-fleshed *A. chinensis* cv ‘HY’ have good potential to be developed and utilized as functional foods and nutraceuticals.

## 1. Introduction

Kiwifruit (*Actinidia*) is one of the most popular fruits around the world due to its unique nutritional value and attractive taste [1,2,3]. To date, more than 70 species of kiwifruits have been identified around the world, and the most commercially valuable species are *A. chinensis* and *A. deliciosa* [3]. In addition, kiwifruits can be categorized into three types based on the flesh color, e.g., yellow, green, and red, and the red-fleshed kiwifruits have more bioactive polyphenols than green-fleshed kiwifruits [4,5]. In fact, kiwifruit is rich in polyphenols (e.g., chlorogenic acid, catechin, procyanidin B1, procyanidin B2, and quercetin), which can contribute to its various health benefits, e.g., antioxidant capacity, the anti-inflammatory effect, the anti-hyperglycemic effect, and the anti-obesity effect [3,4,6]. So, the consumption of kiwifruits can be beneficial for the management and prevention of certain diseases, e.g., oxidative damage and metabolic disorders.

Usually, fruit thinning is a common practice employed to enhance the fruit quality and yield of kiwifruits during the growing period [7,8], and about 30–50% of young kiwifruits will be thinned. These thinned young kiwifruits are always discarded in orchards, resulting in severe environmental pollution and wastage of natural resources. In fact, these unripe young kiwifruits are also rich in nutrients and bioactive compounds. A recent study has revealed that total phenolics, flavonoids, and flavanols in thinned young kiwifruits are significantly higher than those of mature kiwifruits [7], suggesting that unripe kiwifruits have good potential to be developed and utilized as functional foods and nutraceuticals. Nevertheless, the applications of thinned young kiwifruits and their bioactive compounds in the food and functional food industry are still limited. Therefore, to promote the potential applications of thinned young kiwifruits as healthy value-added products in the functional food industry, it is necessary to establish environmentally friendly and efficient approaches for the extraction and preparation of bioactive polyphenols from thinned young kiwifruits.

Generally, the type and content, as well as the biological activities, of phenolic compounds from kiwifruits are affected by different extraction methods [4]. Currently, the conventional organic solvent extraction (CSE) is widely applied for the extraction of phenolic compounds [9]. However, this method always has some drawbacks, such as low phenolic yield, long extraction time, and high solvent consumption [4,9]. To overcome these drawbacks, several new extraction techniques, such as ultrasound-assisted ethanol extraction (UAEE) and subcritical water extraction (SWE), have been explored for the extraction of phenolic compounds from kiwifruits [2,10,11]. In particular, an efficient UAEE method has been developed for the extraction of antioxidants (e.g., phenolics and flavonoids) from kiwifruits [2]. Indeed, the ABTS radical scavenging ability of kiwifruit extract prepared by UAEE is about 18.5% higher than that of CSE. Nevertheless, the conventional organic solvents (e.g., ethanol and acetone) are still utilized during ultrasound-assisted extraction, which also possesses evident drawbacks, such as being environmentally unfriendly and toxic [9]. Recently, the growing focus on ecological sustainability and the green economy has attracted increasing interest in deep eutectic solvents (DESs) as a novel solvent for the extraction of phenolic compounds from plants [12]. As a green solvent, DES has the advantages of cost-effectiveness, low toxicity, biodegradability, and high dissolution ability [12,13,14], demonstrating that DES can be applied as a promising environmentally friendly alternative to traditional organic solvents. Therefore, the combined use of ultrasound and DES can be a promising choice for the green and efficient extraction of phenolic compounds from kiwifruits. Nevertheless, until now, the potential applicability of ultrasound-assisted deep eutectic solvent extraction (UADE) for the preparation of bioactive phenolic compounds from thinned young kiwifruits has never been studied.

Therefore, this study aimed to explore the potential applicability of UADE for the extraction of polyphenolics from thinned young kiwifruits. Furthermore, the phenolic profiles, antioxidant activities, anti-inflammatory activities, and inhibitory effects against digestive enzymes of polyphenolic-rich extracts from thinned young kiwifruits obtained by CSE, UAEE, and UADE were investigated and compared.

## 2. Materials and Methods

### 2.1. Chemicals and Materials

Methanol, ethanol, glycerol, choline chloride, and acetonitrile were obtained from ALADDIN-E (Shanghai, China). DPPH, ABTS, LPS, MTT, Griess reagent, and DMEM medium were obtained from Sigma-Aldrich (St. Louis, MO, USA). Gallic acid (GA), protocatechuic acid (PA), chlorogenic acid (CHL), neochlorogenic acid (NCHL), catechin (Ca), epicatechin (EC), procyanidin B1 (PB1), procyanidin B2 (PB2), quercetin 3-O-glucoside (QGlu), and quercetin 3-O-rhamnoside (QRha) were obtained from Madsen Technology Co., Ltd. (Chengdu, China). All other chemicals were of analytical grade.

Young fruits of red-fleshed *A. chinensis* cv ‘HY’ were thinned 40 days after fruit-set, from trees planted in Deyang Kiwifruit Planting Base, Sichuan, China (GPS coordinate 104°9′17″ E, 31°23′47″ N). The thinned young kiwifruits were thoroughly washed with water. Subsequently, the thinned young kiwifruits were freeze-dried by a freeze-dryer for 48 h. The freeze-dried kiwifruits were further processed into a fine powder using a pulverizer, and then sieved through a 120 mm sieve. The obtained powder was tightly sealed in airtight plastic bags and stored at −20 °C for subsequent experimental use.

### 2.2. Ultrasound-Assisted Deep Eutectic Solvent Extraction (UADE) of Phenolic Compounds from Thinned Young Kiwifruits

#### 2.2.1. Preparation of Deep Eutectic Solvent

Choline chloride (ChCl) and glycerol (Gly) were employed as the two chemicals for the synthesis of deep eutectic solvents (DESs), which were prepared based on the procedure established by Luo et al. [15]. In brief, the DES was prepared by mixing ChCl and Gly in a molar ratio of 1:2, followed by the addition of 20% of water (*v*/*v*) under gentle stirring. The mixture was then sonicated by an ultrasonic cleaning cell until a homogenous solution was obtained.

#### 2.2.2. Optimization of UADE Conditions

The thinned young kiwifruit powder (1.0 g) was mixed with the DES solution, and then extracted using a JY92-IIN ultrasonic processor (Ningbo Scientz Biotechnology Co., Ltd., Ningbo, China). Afterward, the extracted mixture was then subjected to centrifugation (6000× *g*, 15 min), and the supernatant was filtered for further analysis. The polyphenolic-rich extract of thinned young kiwifruits prepared by UADE was coded as UDE. To optimize the conditions of UADE, the effects of the liquid–solid ratio (20:1, 30:1, 40:1, 50:1, and 60:1 mL/g), the ultrasonic power (350, 400, 450, 500, and 550 W), the ultrasonic time (10, 15, 20, 25, and 30 min), and the water content in DES solution (20, 30, 40, 50, and 60%) on the yields of total phenolic compounds from thinned young kiwifruits were investigated. Furthermore, three main variables were selected and further optimized using response surface methodology (RSM). Three independent variables, namely the water content in DES solution (20, 30, and 40%), the liquid–solid ratio (40:1, 50:1, and 60:1 mL/g), and the ultrasonic time (20, 25, and 30 min), were optimized by a three-level Box–Behnken design (BBD) with four factors. The obtained data were analyzed using Design Expert software (version 11) to assess the significance of the model through analysis of variance (ANOVA). The experimental data were subjected to analysis using a second-order polynomial model as follows:

(1) $$\gamma =\beta_{0\;}+\sum \beta_{i}X_{j}+\sum \beta_{\mathrm{ii}}X_{i}^{2}+\sum \beta_{\mathrm{ij}}X_{i}X_{j}$$

where the predicted response is represented by γ, while X_i_ and X_j_ represent different variables. The intercept, and linear, quadratic, and interaction regression coefficients are denoted by β_0_, β_i_, β_ii_, and β_ij_, respectively.

### 2.3. Ultrasound-Assisted Ethanol Extraction (UAEE) of Phenolic Compounds from Thinned Young Kiwifruits

The extraction of total polyphenolics from thinned young kiwifruits by UAEE was conducted according to a previously established procedure by Mai et al. [2]. In brief, 68% of ethanol was added into the thinned young kiwifruit powder (1:20 g/mL), and then subjected to ultrasonic extraction (420 W and 30 min). The extracted mixture was then centrifuged (6000× *g*, 15 min), and the supernatant was filtered for further analysis. The polyphenolic-rich extract of thinned young kiwifruits prepared by UAEE was coded as UEE.

### 2.4. Conventional Organic Solvent Extraction (CSE) of Phenolic Compounds from Thinned Young Kiwifruits

The extraction of phenolic compounds from thinned young kiwifruits by CSE was also conducted according to a previously established procedure by Mai et al. [2]. In brief, 68% of ethanol was added into the thinned young kiwifruit powder (1:10 g/mL), and then subjected to an air bath at 42 °C for a duration of 30 min. The extracted mixture was then subjected to centrifugation (6000× *g*, 15 min), and the supernatant was filtered for further analysis. The polyphenolic-rich extract of thinned young kiwifruits prepared by CSE was coded as EE.

### 2.5. Determination of Total Polyphenolics in the Thinned Young Kiwifruit Extracts

The determination of total phenolic content (TPC) in the thinned young kiwifruit extracts prepared by different extraction methods was conducted by using a modified Folin–Ciocalteu colorimetric method by Mai et al. [2]. TPC in the kiwifruit extracts was expressed as mg GAE/g DW, representing gallic acid equivalent per gram of kiwifruit dry weight. The total flavonoid content (TFC) in the kiwifruit extracts was detected by utilizing the AlCl_3_-based colorimetric method established by Mai et al. [2]. TFC in the kiwifruit extracts was expressed as mg RE/g DW, indicating the rutin equivalent per gram of kiwifruit dry weight. In addition, the total procyanidin content (TPAC) in the kiwifruit extracts was quantified by utilizing a modified vanillin-sulfuric acid colorimetric method by Lin et al. [16]. TPAC in the kiwifruit extracts was expressed as mg CE/g DW, representing the catechin equivalent per gram of kiwifruit dry weight.

### 2.6. Identification of Phenolic Compounds in the Thinned Young Kiwifruit Extracts by UPLC-MS/MS

High-resolution UPLC-MS/MS analysis was carried out by utilizing a Thermo Scientific SII system (San Jose, CA, USA) following our previously established method [2]. Briefly, a Hypersil GOLD column (2.1 × 100 mm, 1.9 µm) was utilized. The mobile phases were 0.5% formic acid–water solution (solvent A) and acetonitrile (solvent B). The high-resolution Q-Exactive Focus mass spectrometer was operated in negative mode, and the orbital trap was set to scan a range of *m*/*z* 100–1000. Data acquisition was performed using Xcalibur 5.0 software (Thermo Scientific, San Jose, CA, USA). To analyze individual phenolics in the thinned young kiwifruit extracts, the parent ions were compared with the MassBank database (https://massbank.eu/MassBank/, accessed on 30 June 2023) and previous literature, as well as several authentic standards (GA, CHL, NCHL, Ca, EC, PB1, PB2, QGlu, and QRha). The deviation between the calculated and observed *m*/*z* values was less than 5 ppm during the identification of individual compounds.

### 2.7. Quantification of Major Phenolic Compounds in the Thinned Young Kiwifruit Extracts

The contents of major phenolics in the kiwifruit extracts prepared by different extraction methods were measured using an Agilent 1260 II HPLC system (Palo Alto, CA, USA) according to our previously established approach [1]. A ZORBAX Eclipase XDB-C18 (4.6 mm × 250 mm, 5 µm) was utilized, and solvent A and solvent B used in this study were 0.5% (*v*/*v*) of acetic acid solution and acetonitrile, respectively. Both hydroxybenzoic acids and flavan-3-ols were measured at 280 nm, and hydroxycinnamic acids and flavonols were measured at 320 nm and 360 nm, respectively. To quantify the phenolic compounds in the kiwifruit extracts, ten standards were used, including four phenolic acids (GA, PA, CHL, and NCHL), four flavan-3-ols (Ca, EC, PB1, and PB2), and two flavonols (QGlu and QRha). The content of individual phenolic compound was expressed in mg/g kiwifruit dry weight (mg/g DW).

### 2.8. Evaluation of Antioxidant Capacities of the Thinned Young Kiwifruit Extracts

To understand the antioxidant capacities of the thinned young kiwifruit extracts prepared by different extraction methods, the ABTS radical scavenging ability assay, the DPPH radical scavenging ability assay, the hydroxyl radical (OH) scavenging ability assay, and the ferric-reducing antioxidant power (FRAP) assay were carried out in this study as previously described [1]. For the determination of ABTS radical scavenging ability, the absorbance was measured at 734 nm, and the ABTS radical scavenging ability was exhibited as micromolar Trolox equivalent per gram of kiwifruit dry weight (µmol Trolox/g DW). For the determination of DPPH radical scavenging ability, the absorbance was recorded at 517 nm, and the DPPH antioxidant capacity was also exhibited as µmol Trolox/g DW. For the determination of OH radical scavenging ability, the absorbance was detected at 510 nm, and the OH antioxidant capacity was also exhibited as µmol Trolox/g DW. For the determination of FRAP, the absorbance was measured at 593 nm, and the FRAP antioxidant capacity was also exhibited as µmol Trolox/g DW.

### 2.9. Evaluation of Inhibitory Effects of the Thinned Young Kiwifruit Extracts against α-Glucosidase and Pancreatic Lipase

The inhibitory effects of the thinned young kiwifruit extracts prepared by different extraction methods against α-glucosidase were assessed according to a previous procedure [1]. Acarbose tablets (each tablet contains 50 mg of acarbose; Bayer HealthCare Company Ltd., Beijing, China) were employed as a positive control for the α-glucosidase inhibition assay. The inhibitory effects were exhibited as the percentage of inhibition rate (%), and the IC_50_ values (μg kiwifruit dry weight per mL, μg/mL) were calculated by establishing a logarithmic regression curve. In addition, the measurement of pancreatic lipase activity inhibition was also conducted according to a previous procedure [1]. Orlistat capsules (each capsule contains 0.12 g of orlistat; Chongqing Pharscin Pharmaceutical Co., LTD., Chongqing, China) were employed as a positive control in this study. The inhibitory effects were also exhibited as the percentage of inhibition rate (%), and the IC_50_ values (mg kiwifruit dry weight/mL, mg/mL) were calculated by establishing a logarithmic regression curve.

### 2.10. Evaluation of In Vitro Anti-Inflammatory Activities of the Thinned Young Kiwifruit Extracts

The anti-inflammatory effects of the thinned young kiwifruit extracts prepared by different extraction methods were evaluated using an LPS-induced RAW 264.7 cell model as previously reported [17]. In brief, the impact of the thinned young kiwifruit extracts on the cell viability of RAW 264.7 cells was evaluated by the MTT method, and different concentrations (6.25–50.00 µg kiwifruit dry weight/mL, µg/mL) of the kiwifruit extracts were tested. In addition, the RAW 264.7 macrophages were cultured overnight in 96-well plates at 37 °C with 5% CO_2_. The culture medium was then replaced with LPS (1 µg/mL) in a volume of 100 µL per well, except for the blank group. After incubating for 24 h, various concentrations (12.50–50.00 µg kiwifruit dry weight/mL, µg/mL) of the kiwifruit extracts were added and incubated for an additional 24 h. The supernatant (50.0 μL) was mixed with Griess I (50.0 μL) and Griess II (50.0 μL) reagents at room temperature, and the absorbance was measured at 540 nm. NaNO_2_ was employed as a reference standard to determine the concentration of NO.

### 2.11. Statistical Analysis

Data analysis was conducted using IBM SPSS Statistics 26 software. The results were expressed as mean ± standard deviation (n ≥ 3). Statistical analysis was performed by one-way ANOVA or two-tailed Student t-test. The threshold for statistical significance was set at *p* < 0.05. Additionally, Pearson’s correlation coefficient was calculated using Origin 2022 software to evaluate potential correlations.

## 3. Results and Discussion

### 3.1. Optimal Conditions of UADE for the Extraction of Phenolic Compounds from Thinned Young Kiwifruits

#### 3.1.1. Optimal Conditions from the Single-Factor Experiment

**Figure 1:**
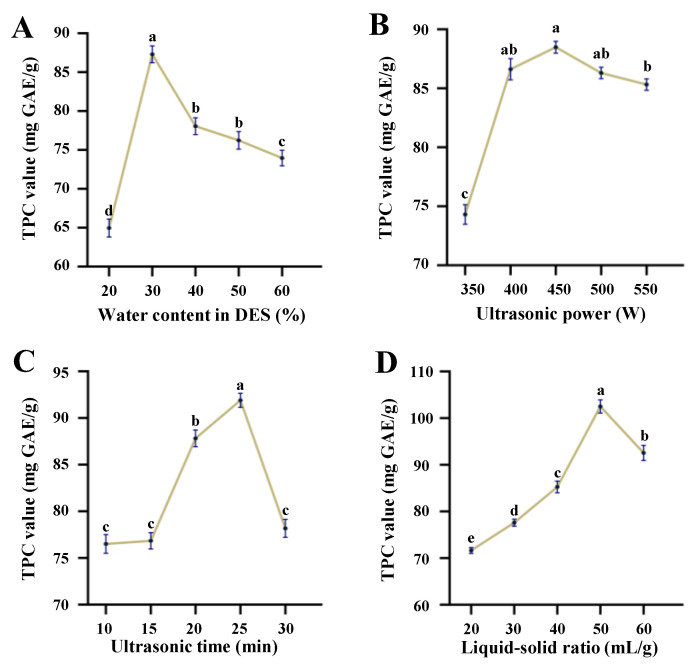
Effects of water content in DES solution (**A**), ultrasonic power (**B**), ultrasonic time (**C**), and liquid–solid ratio (**D**) on the yield of total phenolics extracted from of thinned young kiwifruits by ultrasound-assisted deep eutectic solvent extraction. TPC indicates total phenolic content; different letters (a–e) indicate significant differences at *p* < 0.05 determined by ANOVA.

Generally, the liquid–solid ratio, ultrasonic power, ultrasonic time, and water content in DES solution are important parameters that significantly affect the extraction efficiency of UADE [2,15]. Therefore, to identify the most crucial parameters for the subsequent optimization, the single-factor experiments were conducted first. Figure 1A shows the effect of water content in the DES solution on the yield of TPC extracted from thinned young kiwifruits. Results showed that a noteworthy increase in TPC values was observed when the water content in the DES solution increased from 20% to 30%. However, the TPC values decreased significantly when the water content further increased from 30% to 60%. The addition of an appropriate amount of water in DES solution could effectively reduce the viscosity and improve the extraction efficiency of phenolic compounds [13]. Nevertheless, the excessive addition of water in the DES solution could destroy the hydrogen-bonding interactions, resulting in the decrease in extraction efficiency of phenolic compounds [18]. Therefore, based on the obtained results, the water content of 30% in the DES solution was ultimately selected for subsequent single-factor experiments. In addition, as displayed in Figure 1B, an increase in ultrasonic power from 350 W to 550 W increased TPC values. The maximum TPC value was obtained at an ultrasonic power of 450 W, beyond which the TPC values started decreasing. Generally, the increase in ultrasonic power could lead to a high hydrodynamic force, which resulted in a great disruption of the cell wall and facilitated the extraction process [19]. However, it was also observed that the excessive ultrasonic power could cause an increase in the number of bubbles formed during the cavitation, resulting in a decrease in the efficiency of energy transferred to the solvent [15]. Nevertheless, the TPC values obtained at the ultrasonic powers of 400, 450, and 500 W showed no significant differences. Furthermore, as illustrated in Figure 1C, the TPC values remarkably improved from 10 to 25 min, and then decreased from 25 to 30 min. An increase in the ultrasonic time could promote the extraction efficiency of polyphenols. However, the prolonged ultrasonic time may lead to the degradation of bioactive antioxidant-like compounds in the kiwifruit extracts, eventually resulting in a decrease in extraction efficiency [20]. Finally, the effect of the liquid–solid ratio on the yield of TPC extracted from thinned young kiwifruits was investigated. The results showed that the TPC values increased with increasing liquid–solid ratios from 20:1 to 50:1 (mL/g) (Figure 1D), and then slightly decreased from 50:1 to 60:1 (mL/g). Therefore, after the single-factor experimental optimization, the optimal conditions, including water content in DES solution, ultrasonic power, ultrasonic time, and liquid–solid ratio, were determined to be 30%, 450 W, 25 min, and 50 mL/g, respectively.

#### 3.1.2. Optimal Conditions from the Box-Behnken Design

After performing the single-factor experiments, the liquid–solid ratio, water content in DES solution, and ultrasonic time were determined as essential parameters that significantly affect the extraction efficiency of phenolic compounds from thinned young kiwifruits. Thus, these three variables were selected as independent factors for the RSM optimization. The BBD design resulted in 17 experiments, the outcomes of which are summarized in Table 1. The TPC values obtained from these experiments varied from 68.74 to 106.57 mg GAE/g. In addition, multiple regression analysis was employed to analyze the experimental data, and a second-order polynomial equation was generated to express the relationship between the independent variables and the responses as follows:

(2) $$Y=104.69+8.11X_{1}+4.97X_{2}-5.00X_{3}+4.06X_{1}X_{2}+1.89X_{1}X_{3}-1.13X_{2}X_{3}-12.70X_{1}^{2}-12.90X_{2}^{2}-6.35X_{3}^{2}$$

where Y is the TPC value, and X_1_ is the water content in DES solution (%), X_2_ is the liquid-solid ratio (mL/g), and X_3_ is the ultrasonic time (min).

Furthermore, the statistical significance of the second-order response surface model was assessed by one-way ANOVA analysis, and the results are displayed in Table 2. The fitted model had a high *F*-value (131.72) and an extremely low *p*-value (<0.0001). Therefore, we could conclude that the fitted model was significant [21]. Conversely, the *F*-value of lack of fit was 1.15 and the *p*-value of lack of fit was 0.4305, indicating that the fitted model could be used to accurately predict the response value [21,22]. Additionally, a small coefficient variation indicates a small degree of variation in the response value, resulting in high accuracy and reliability of the fitted model [21,22]. Therefore, the low coefficient variation (*C.V.*, 1.69%) indicated a good fit and predictive accuracy of the response surface regression model. Furthermore, the coefficient of determination (R^2^) and the adjusted R^2^ were measured to be 0.9941 and 0.9866, respectively, which further confirmed the reliability of this fitted model [21,22]. Moreover, the linear parameters (X_1_, X_2_, and X_3_), the interactive parameters (X_1_X_2_ and X_1_X_3_), and the quadratic parameters (X_1_^2^, X_2_^2^, and X_3_^2^) were all significant. Collectively, it was inferred that the regression model was reasonable.

**Figure 2:**
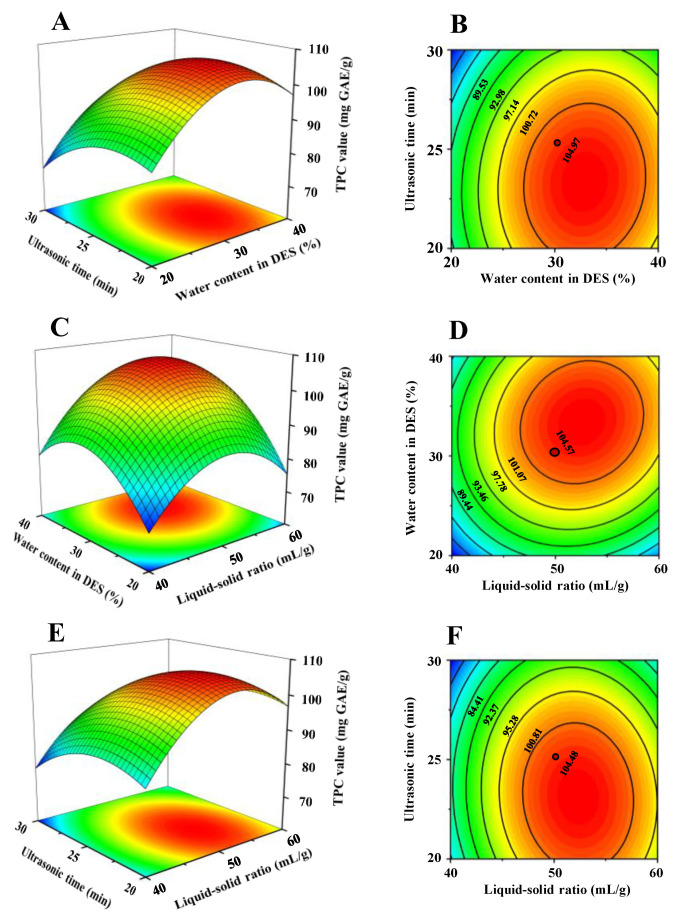
Three-dimensional response surface plots (**A**,**C**,**E**) and 2D contour plots (**B**,**D**,**F**) of ultrasound-assisted deep eutectic solvent extraction. (**A**–**F**) indicate interactions among ultrasonic time, water content in DES, and liquid–solid ratio, respectively.

Figure 2 shows the 2D/3D plots of the fitted model. As shown in Figure 2, it could be inferred that the water content in DES solution and the liquid–solid ratio exhibited more positive impacts on the yields of total phenolic compounds from thinned young kiwifruits than those of ultrasonic time. According to the ANOVA results and the response surface plots, the interaction effects between the water content in DES solution and the liquid-solid ratio (X_1_X_2_) and the water content in the DES solution and the ultrasonic time (X_1_X_3_) were significant. Moreover, a maximum value (107.25 mg GAE/g) of TPC extracted from thinned young kiwifruits could be obtained under the predicted optimal conditions as follows: 31.94% of water content in DES solution, liquid–solid ratio of 51.45:1 mL/g, and ultrasonic time of 23.05 min. Nevertheless, regarding the practice in the actual processing procedure, the validation experiment was performed under the following conditions: water content of 32% in DES solution, liquid–solid ratio of 50:1 mL/g, and ultrasonic time of 23 min. Under these extraction conditions, the yield of TPC obtained from thinned young kiwifruits by UADE was 105.37 ± 1.2 mg GAE/g (n = 3), which was inconsistent with the predicted value (107.25 mg GAE/g).

Furthermore, compared with the CSE and UAEE methods, the optimized UDAE method had a higher extraction rate of total phenolic compounds, while having a much shorter extraction time. The TPC value of UDE (105.37 ± 1.2 mg GAE/g) extracted by UDAE was about 7.3 times than that of EE extracted by CSE (14.51 ± 0.26 mg GAE/g DW), and about 2.4 times than that of UEE extracted by UAEE (43.85 ± 1.17 mg GAE/g DW). The TFC (about 19.53 ± 0.35 mg RE/g DW) and TPAC (about 9.85 ± 0.06 mg CE/g DW) values of UDE were also much higher than those of UEE (9.83 ± 0.26 mg RE/g DW and 4.71 ± 0.17 mg CE/g DW) and EE (2.76 ± 0.17 mg RE/g DW and 1.61 ± 0.08 mg CE/g DW), respectively. In fact, the ultrasound can generate a high-intensity physical effect that disrupts plant cell walls and cell membranes, facilitating the release of polyphenols [23]. In addition, the strong chemisorption of DES solution to polyphenol molecules occurs through hydrogen bonding, van der Waals forces, and other interactions, thereby improving the extraction efficiency of polyphenols [24]. Therefore, the UDAE method possessed a higher extraction efficiency of polyphenols from thinned young kiwifruits than that of CSE and UAEE methods, and the UDAE method could be applied as an efficient and green technique to prepare polyphenolics from thinned young kiwifruits. Furthermore, compared with mature kiwifruits of different species [1,2], the thinned young fruits of red-fleshed *A. chinensis* cv ‘HY’ had a much higher content of total phenolics; this finding is similar to that of previous studies, in which unripe kiwifruits exhibited higher contents of total phenolics and total flavonoids than those of mature kiwifruits [7,25]. Results indicated that the thinned young fruits of red-fleshed *A. chinensis* cv ‘HY’ could be used as natural resources of polyphenolics.

### 3.2. Identification and Quantification of Major Phenolic Compounds in the Thinned Young Kiwifruit Extracts

**Figure 3:**
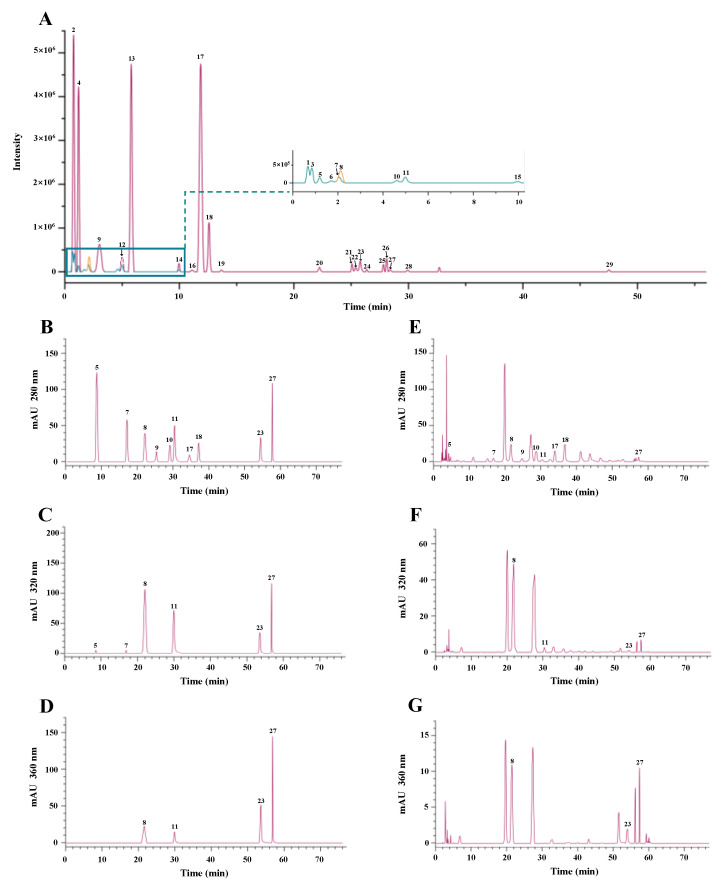
UPLC-MS/MS extracted ions chromatogram (**A**) and HPLC chromatograms of mixed standards (**B**–**D**) and the thinned young kiwifruit extract (**E**–**G**). Compounds **1**–**29** are the same as in Table 3.

High-resolution UPLC-MS/MS analysis was carried out to characterize the major phenolic compounds in the kiwifruit extracts. As shown in Figure 3, a total of 29 compounds were tentatively identified from the thinned young fruits of red-fleshed *A. chinensis* cv ‘HY’, and their retention times, formulas, calculated *m*/*z*, and observed *m*/*z* are summarized in Table 3. Based on the previous literature, MassBank database, and several authentic standards, these compounds were tentatively identified as ascorbic acid, citric acid, quinic acid, GA, sinapic acid, syringic acid, vanillic acid, CHL, NCHL, 3-p-coumaroylquinic acid, PA-O-hexoside, caffeic acid-O-hexoside, Ca, EC, gallocatechin, PB1, PB2, procyanidin trimer C1, fraxin, astilbin, hyperoside, pinostrobin, apigenin, hesperidin, arbutin, kaempferol, rutin, QGlu, and QRha. Among these, ascorbic acid, citric acid, quinic acid, GA, syringic acid, vanillic acid, CHL, NCHL, 3-p-coumaroylquinic acid, PA-O-hexoside, caffeic acid-O-hexoside, Ca, EC, PB1, PB2, procyanidin trimer C1, hyperoside, apigenin, kaempferol, rutin, QGlu, and QRha have also been found in kiwifruits of various species [1,2,3,7,26,27,28].

Furthermore, to gain a better understanding of the effect of different methods on the contents of individual phenolics in the kiwifruit extracts, HPLC-DAD analysis was conducted to quantify their contents. According to the high-resolution UPLC-MS/MS analysis, ten commercially available phenolic standards, including GA, PA, NCHL, CHL, Ca, EC, PB1, PB2, QGlu, and QRha, were quantified in the kiwifruit extracts prepared by UDAE, UAEE, and CSE methods. Figure 3 displays the HPLC profiles of mixed standards and the representative extract of thinned young kiwifruits. The calibration data of the 10 compounds and their contents in different kiwifruit extracts are summarized in Table 4. Results revealed that the content of total flavan-3-ols (Ca, EC, PB1, and PB2) was the highest among the quantified phenolic compounds in the kiwifruit extracts prepared by different methods, followed by that of total phenolic acids (GA, PA, CHL, and NCHL), and the lowest was that of total flavonols (QGlu and QRha). In particular, PB2 was determined as the most abundant phenolic compound among these ten phenolic compounds in the thinned young kiwifruit extracts prepared by different extraction methods, and its abundance was much higher than that of its isomer (PB1). NCHL was determined as the most abundant phenolic acid in the thinned young kiwifruit extracts prepared by different extraction methods, and its abundance was much higher than that of its isomer (CHL). In addition, results revealed that the contents of individual phenolic compounds were obviously affected by different extraction methods, similar to the results of TPC values. The highest content (15.067 ± 1.143 mg/g DW) of phenolic compounds was found in UDE prepared by UADE, followed by lower UEE prepared by UAEE (6.122 ± 0.074 mg/g DW), and the lowest was EE prepared by CSE (2.218 ± 0.271 mg/g DW). Results further confirmed that the UADE method exhibited a higher extraction efficiency of polyphenols than that of CSE and UAEE methods, similar to previous studies that showed the UADE method could be a highly efficient method for the extraction of polyphenolics from natural resources [15,29,30]. Furthermore, the content of total phenolics measured by HPLC analysis was much lower than that of the Folin–Ciocalteu colorimetric method, which was similar to previous studies [1,2,7]. This was probably due to the fact that the HPLC analysis only focused on ten phenolic compounds, and the accuracy of the Folin–Ciocalteu colorimetric method could be influenced by the presence of amino acids, reducing sugars, and ascorbic acids in the crude extract [31]. Notably, the content of PB2 in UDE (4.799 ± 0.128 mg/g DW) was about 2.9 and 7.5 times higher than that of UEE (1.635 ± 0.081 mg/g DW) and EE (0.644 ± 0.057 mg/g DW), respectively. Concerning the total content of phenolic acids, UDE was about 2.7 and 9.2 times higher than that of UEE and EE, respectively. The content of total flavonols in UDE was about 2.8 and 8.7 times higher than that of UEE and EE, respectively. Collectively, UADE, as a green extraction method, is more advantageous for the extraction of polyphenolics from thinned young fruits of red-fleshed *A. chinensis* cv ‘HY’ than conventional methods.

### 3.3. Comparison of Antioxidant Capacities of the Thinned Young Kiwifruit Extracts

**Figure 4:**
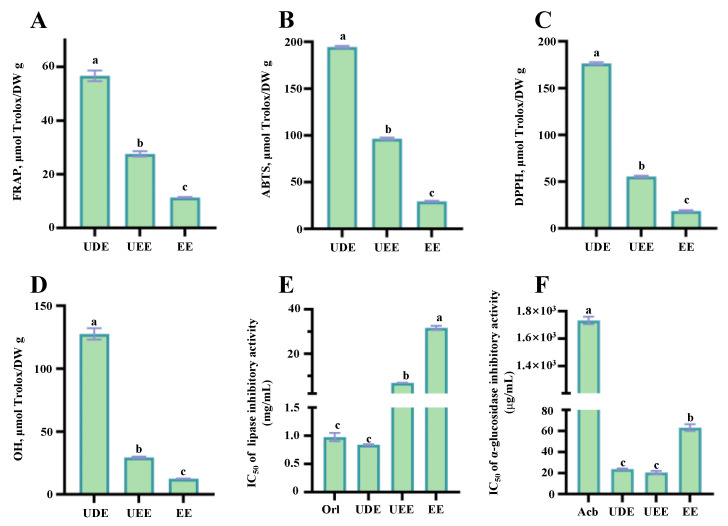
Antioxidant capacities and inhibitory effects on digestive enzymes of the thinned young kiwifruit extracts: (**A**), the ferric-reducing antioxidant power; (**B**), the ABTS radical scavenging ability; (**C**), the DPPH radical scavenging ability; (**D**), the hydroxyl radical scavenging ability; (**E**), the inhibitory effect against pancreatic lipase; (**F**), the inhibitory effect against α-glucosidase. UDE, the thinned kiwifruit extract prepared by ultrasound-assisted deep eutectic solvent extraction; UEE, the thinned kiwifruit extract prepared by ultrasound-assisted ethanol extraction; EE, the thinned kiwifruit extract prepared by conventional organic solvent extraction; different letters (a–c) indicate statistically significant differences (*p* < 0.05).

Oxidative stress induced by free radicals plays a critical role in the pathophysiology of various diseases, and the dietary consumption of polyphenolic-rich kiwifruits can protect against oxidative stress [32]. In fact, a large number of studies have revealed that kiwifruits exhibit remarkable free radical scavenging abilities, which are positively associated with their abundant polyphenols [3,4]. A recent study has also revealed that thinned young kiwifruits possess obviously higher antioxidant activities than mature kiwifruits [7]. Nevertheless, the impact of different extraction methods on the antioxidant activity of thinned young kiwifruits was still unclear. Therefore, the free radical scavenging abilities (e.g., ABTS, DPPH, and OH free radicals) and ferric-reducing antioxidant powers of the kiwifruit extracts prepared by UADE, UAEE, and CSE were studied and compared. As shown in Figure 4, it could be observed that the antioxidant capacities of UDE prepared by the UADE method determined by different assays were significantly higher than those of UEE and EE, indicating that the UADE method could efficiently extract antioxidants from thinned young kiwifruits. In detail, as displayed in Figure 4A, the FRAP values of different kiwifruit extracts (UDE, UEE, and EE) ranged from 11.35 ± 0.18 μmol Trolox/g DW (EE) to 56.67 ± 2.01 μmol Trolox/g DW (UDE), and the highest FRAP value was observed in UDE. Furthermore, as shown in Figure 4B–D, the ABTS, DPPH, and OH free radical scavenging abilities ranged from 29.45 ± 0.62 μmol Trolox/g DW to 194.52 ± 1.16 μmol Trolox/g DW, from 18.57 ± 0.84 μmol Trolox/g DW to 176.41 ± 1.37 μmol Trolox/g DW, and from 12.57 ± 0.12 μmol Trolox/g DW to 127.63 ± 4.53 μmol Trolox/g DW, respectively, and the highest free radical scavenging abilities were also determined in UDE. In fact, the highest antioxidant capacity of UDE might be closely correlated to its highest content of total phenolic compounds, similar to previous studies that showed polyphenols were the major contributors to the antioxidant activity of kiwifruits [1,5,7,26,33,34].

**Figure 5:**
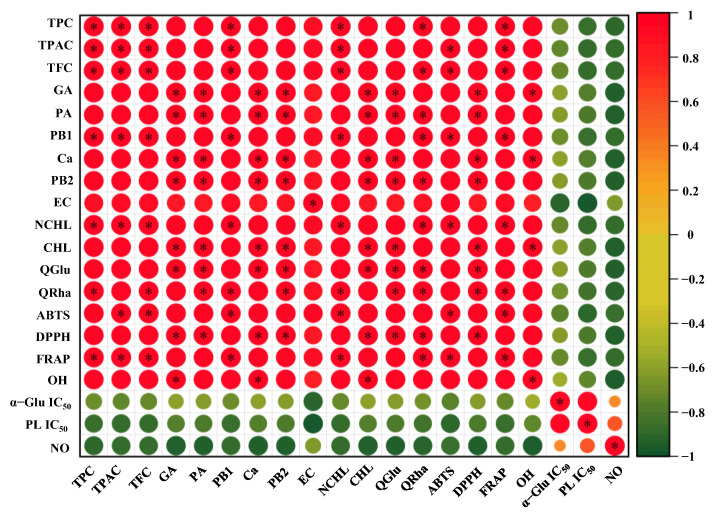
Pearson correlation matrix of phenolics, antioxidant capacities, inhibitory effects on digestive enzymes, and anti-inflammatory effects. The correlation coefficients are proportional to the circular size and color intensity; * Stands for significant correlation at *p* < 0.05.

Furthermore, according to Pearson analysis (Figure 5), the values of ABTS, DPPH, and OH free radical scavenging abilities, as well as FRAP, showed strongly positive correlations with the values of TPC (r, 0.979–0.999), TFC (r, 0.969–0.999), and TPAC (r, 0.971–0.999). In detail, both TFC and TPAC showed significant (*p* < 0.05) positive correlations with ABTS, and TPC, TFC, and TPAC exhibited significant (*p* < 0.05) positive correlations with FRAP, similar to previous studies [7,34,35]. Additionally, PB1 and NCHL demonstrated significantly positive correlations with the ABTS free radical scavenging ability. GA, PA, Ca, PB2, CHL, QGlu, and QRha exhibited significantly positive correlations with the DPPH free radical scavenging ability. Furthermore, PB1, NCHL and QRha displayed significantly positive correlations with the FRAP, and GA, Ca, and CHL demonstrated significantly positive correlations with the OH free radical scavenging activity. Collectively, these results suggest that the UADE method could be applied as an efficient technique for the extraction of antioxidants from thinned young kiwifruits, and the thinned young fruits of red-fleshed *A. chinensis* cv ‘HY’ could be developed as natural antioxidants in the functional food, pharmaceutical, and cosmetics industries.

### 3.4. Comparison of Inhibitory Effects on Digestive Enzymes of the Thinned Young Kiwifruit Extracts

Inhibition of the pancreatic lipase, a key enzyme in lipid metabolism, is crucial for effectively preventing triglyceride digestion in patients with hypercholesterolemia [36]. Previous studies have shown that kiwifruit polyphenols have a potent inhibitory effect on the enzymatic activity of pancreatic lipase [1,25,37]. Nevertheless, the inhibitory effect of polyphenolics extracted from thinned young kiwifruits against pancreatic lipase is still unclear, and whether the extraction methods affect their inhibitory rates is also unknown. Figure 4E shows the IC_50_ values of different kiwifruit extracts (UDE, UEE, and EE) against the enzymatic activity of pancreatic lipase. Obviously, different extraction methods significantly affected the inhibitory rates of kiwifruit extracts against pancreatic lipase. In detail, among different kiwifruit extracts (UDE, UEE, and EE), the strongest inhibitory effect against pancreatic lipase was observed in UDE (IC_50_ = 0.84 mg/mL), which was also stronger than those of different species of mature kiwifruits (about 3.12 mg/mL–7.44 mg/mL) [1]. In addition, the IC_50_ value of UDE against pancreatic lipase was similar to that of the commercially available orlistat, indicating that UDE exhibited a remarkable inhibitory effect on pancreatic lipase. Conversely, the IC_50_ values of UEE and EE were much higher than that of orlistat. Furthermore, as shown in Figure 5, TPC, TFC, and TPAC were negatively correlated with the IC_50_ values of kiwifruit extracts against pancreatic lipase (r, −0.886–−0.864). Previous studies have shown that polyphenolics play an important role in the inhibition of pancreatic lipase [36,38]. In particular, EC (r, −0.995) was one of the main contributors to the inhibition of pancreatic lipase.

Maintaining a healthy blood glucose level is a critical way to manage the progression of diabetes and obesity. Polyphenols can inhibit the enzymatic activity of α-glucosidase, thereby preventing the absorption of excess glucose in the small intestine [39]. Several studies have also revealed that kiwifruit polyphenolics exhibit potential inhibitory effects on α-glucosidase [1,25]. However, the impact of extraction methods on the inhibitory rate of unripe kiwifruit extracts is still unknown. As shown in Figure 4F. compared with the positive control (acarbose tablet, IC_50_ = 1.76 mg/mL), different thinned young kiwifruit extracts (UDE, UEE, and EE) exhibited stronger inhibitory effects against α-glucosidase, with the IC_50_ values ranging from 23.82 µg/mL to 62.98 µg/mL. The inhibitory effects of different thinned young kiwifruit extracts (UDE, UEE, and EE) against α-glucosidase were also stronger than those of different species of mature kiwifruits (about 9.11 mg/mL–66.73 mg/mL) [1]. Additionally, the inhibitory effect of UDE against α-glucosidase was similar to that of UEE, while significantly higher than that of EE. Furthermore, according to the correlation analysis, TPC, TFC, and TPAC exhibited negative correlations with the IC_50_ values of kiwifruit extracts against α-glucosidase (r, −0.741–−0.708). In particular, EC (r, −0.938) was also found as one of the main contributors to the inhibitory effect against α-glucosidase, probably due to its hydroxyl and phenyl groups [40]. Collectively, these results indicate that polyphenolics extracted from thinned young fruits of red-fleshed *A. chinensis* cv ‘HY’ could be developed as functional ingredients for the prevention and management of hyperglycemia and hyperlipidemia.

### 3.5. Comparison of Anti-Inflammatory Effects of the Thinned Young Kiwifruit Extracts

Accumulating experimental studies have demonstrated that kiwifruit powder and polyphenols extracted from various species of kiwifruits exhibit anti-inflammatory effects [3]. For instance, the freeze-dried mature kiwifruit powder can reduce the nitric oxide (NO) production in lipopolysaccharide (LPS)-stimulated RAW 264.7 cells [41], which can also inhibit overexpression of proinflammatory cytokines (e.g., IL-6 and TNF-α) via mitogen-activated protein kinases pathway in the mice colon. In addition, polyphenol-rich extracts of mature kiwifruits can downregulate the levels of IL-6 and TNF-α in LPS-stimulated human THP-1 monocytes [42]. Nevertheless, the in vitro anti-inflammatory effect of polyphenols extracted from unripe kiwifruits is unclear. Therefore, the anti-inflammatory effects of polyphenols-rich extracts of thinned young kiwifruits prepared by different methods were investigated.

**Figure 6:**
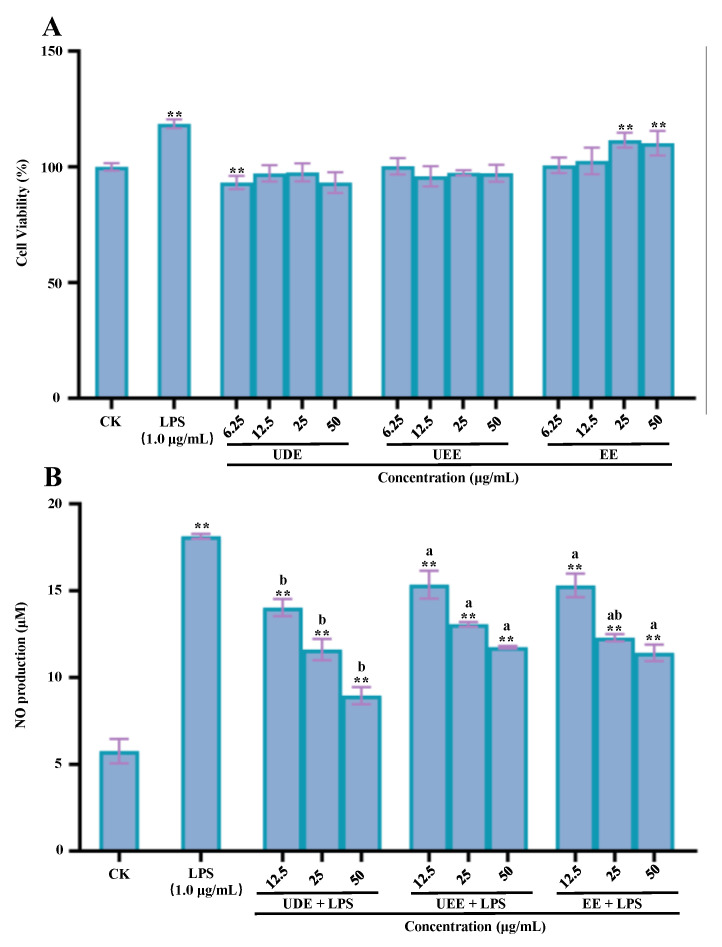
In vitro anti-inflammatory effects of the thinned young kiwifruit extracts: (**A**), the effect of different thinned young kiwifruit extracts on the cell viability of RAW 264.7 cells; (**B**), the effect of different thinned young kiwifruit extracts on the NO production from LPS-stimulated RAW 264.7 cells. UDE, the thinned kiwifruit extract prepared by ultrasound-assisted deep eutectic solvent extraction; UEE, the thinned kiwifruit extract prepared by ultrasound-assisted ethanol extraction; EE, the thinned kiwifruit extract prepared by conventional organic solvent extraction; different letters (a, b) indicate statistically significant differences (*p* < 0.05) among different kiwifruit extracts; significant differences in cell viability and NO production in LPS and kiwifruit extracts vs. control are shown by ** *p* < 0.01.

Figure 6A displays the effects of different thinned young kiwifruit extracts (UDE, UEE, and EE) on the cell viability of RAW 264.7 macrophages. Results revealed that all kiwifruit extracts (UDE, UEE, and EE) exhibited no cytotoxicity effects on RAW 264.7 macrophages at concentrations in the range of 6.25–50.00 μg/mL. Furthermore, as shown in Figure 6B, UDE, UEE, and EE could obviously inhibit the production of NO from LPS-induced RAW macrophages, suggesting that polyphenol-rich extracts of thinned young kiwifruits exhibited remarkable in vitro anti-inflammatory effects. Indeed, among all kiwifruit extracts, UDE prepared by the UADE method exhibited the most significant inhibitory effect on the NO production from RAW 264.7 cells, with an inhibition rate of 50.61% at a concentration of 50.00 μg/mL. Furthermore, UEE and EE displayed similar inhibitory effects on the NO production from RAW 264.7 cells, with the inhibition rates ranging from 35.28% to 37.05%. Additionally, according to the correlation analysis (Figure 5), TPC, TFC, and TPAC exhibited negative correlations with the NO production from LPS-stimulated RAW 264.7 cells, suggesting that polyphenols were the major contributors to the anti-inflammatory effect of thinned young kiwifruits, similar to previous studies [42,43,44,45]. Moreover, GA, PA, CHL, Ca, PB2, QGlu, and QRha also exhibited negative correlations with the NO production, indicating that phenolic acids, flavan-3-ols, and flavonols played crucial roles in the inhibition of NO release. Collectively, polyphenol-rich extracts of thinned young kiwifruits could be developed as functional ingredients for the prevention and management of chronic inflammatory diseases.

## 4. Conclusions

In this study, to promote the potential applications of thinned young kiwifruits, the extraction, characterization, and evaluation of bioactivities of phenolic compounds from thinned young fruits of red-fleshed *A. chinensis* cv ‘HY’ were studied. A green and efficient UADE method for the extraction of phenolic compounds from thinned young kiwifruits was established. The content of major phenolic compounds in UDE extracted by UADE was significantly higher than that in EE extracted by CSE and UEE extracted by UAEE. In addition, the UDE exhibited the strongest antioxidant capacities, anti-inflammatory activities, and inhibitory effects on pancreatic lipase and α-glucosidase among all extracts, which might be closely associated with its highest content of total phenolics. These findings indicate that the UADE method can be utilized as a green and efficient method for extracting bioactive phenolic compounds, and the thinned young fruits of red-fleshed *A. chinensis* cv ‘HY’ can be developed and utilized as functional foods and nutraceuticals.

## Figures and Tables

**Table 1:** BBD with independent factors and observed values for the yields of total phenolics from thinned young kiwifruits by ultrasound-assisted deep eutectic solvent extraction.

Runs	Levels of Independent Factors			Extraction Yields (mg GAE/g DW)
	X_1_ (%)	X_2_ (mL/g)	X_3_ (min)	
1	1 (40)	−1 (40)	0 (25)	78.21
2	−1 (20)	−1 (40)	0 (25)	68.74
3	−1 (20)	0 (50)	1 (30)	71.52
4	0 (30)	0 (50)	1 (30)	103.79
5	−1 (20)	0 (50)	−1 (20)	84.91
6	1 (40)	1 (60)	0 (25)	97.56
7	−1 (20)	1 (60)	0 (25)	71.86
8	0 (30)	1 (60)	1 (30)	83.42
9	1 (40)	0 (50)	1 (30)	90.15
10	0 (30)	1 (60)	−1 (20)	96.07
11	0 (30)	−1 (40)	1 (30)	77.06
12	0 (30)	0 (50)	0 (25)	104.16
13	0 (30)	0 (50)	0 (25)	105.86
14	0 (30)	−1 (40)	−1 (20)	85.18
15	1 (40)	0 (50)	−1 (20)	95.98
16	0 (30)	0 (50)	0 (25)	103.07
17	0 (30)	0 (50)	0 (25)	106.57

X_1_, water content in DES solution; X_2_, liquid/solid ratio; X_3_, ultrasonic time.

**Table 2:** Analysis of the variance of the response surface model for ultrasound-assisted deep eutectic solvent extraction.

	Sum of Squares	df	Mean Square	F-Value	*p*-Value
Model	2720.72	9	302.30	131.72	<0.0001 **
X_1_	526.01	1	526.01	229.20	<0.0001 **
X_2_	197.21	1	197.21	85.93	<0.0001 **
X_3_	199.90	1	199.90	28.69	<0.0001 **
X_1_X_2_	65.85	1	65.85	6.23	0.0011 *
X_1_X_3_	14.29	1	14.29	1.76	0.0413 *
X_2_X_3_	5.13	1	5.13	2.24	0.1785
X_1_^2^	678.58	1	678.58	295.68	<0.0001 **
X_2_^2^	700.95	1	700.95	305.42	<0.0001 **
X_3_^2^	170.05	1	170.05	74.09	0.0002 **
Residual	16.07	7	2.30		
Lack of Fit	7.45	3	2.48	1.15	0.4305
Pure error	8.62	4	2.15		
Corrected total	2736.78	16			
R²	0.9941				
Adjusted R²	0.9866				

* Stands for statical significance (*p* < 0.05); ** Stands for statical extremely significance (*p* < 0.001).

**Table 3:** Tentative identification of compounds in the kiwifruit extract by UPLC-MS/MS.

NO.	Formula	Retention Time (min)	Calculated [M-H]^−^	Observed [M-H]^−^	Error (ppm)	Identified Compounds
1	C_15_H_14_O_7_	0.67	305.06668	305.06729	2.00	Gallocatechin ^a^
2	C_7_H_12_O_6_	0.77	191.05611	191.05518	−4.87	Quinic acid ^ab^
3	C_6_H_8_O_6_	0.80	175.02481	175.02376	−4.74	Ascorbic acid ^ab^
4	C_6_H_8_O_7_	1.19	191.01973	191.01894	−4.14	Citric acid ^ab^
5	C_7_H_6_O_5_	1.49	169.01425	169.0106	−2.07	Gallic acid ^abc^
6	C_11_H_12_O_5_	1.70	223.0612	223.06038	−3.68	Sinapic acid ^a^
7	C_13_H_16_O_9_	2.05	315.07216	315.07217	0.03	Protocatechuic acid-O-hexoside ^ab^
8	C_16_H_18_O_9_	2.11	353.08781	353.0878	−0.03	Neochlorogenic acid ^abc^
9	C_30_H_26_O_12_	2.98	577.13515	577.13519	0.07	Procyanidin B1 ^abc^
10	C_15_H_14_O_6_	4.90	289.07176	289.07172	−0.14	Catechin ^abc^
11	C_16_H_18_O_9_	4.98	353.08781	353.0878	−0.03	Chlorogenic acid ^abc^
12	C_9_H_10_O_5_	5.05	197.04555	197.04498	−2.89	Syringic acid ^ab^
13	C_8_H_8_O_4_	5.78	167.03498	167.03415	−4.97	Vanillic acid ^ab^
14	C_16_H_18_O_8_	9.93	337.092829	337.09283	0.00	3-p-Coumaroylquinic acid ^ab^
15	C_15_H_18_O_9_	9.95	341.08781	341.08771	−0.29	Caffeic acid-O-hexoside ^ab^
16	C_16_H_18_O_10_	11.68	369.08272	369.08273	0.03	Fraxin ^a^
17	C_30_H_26_O_12_	11.74	577.13515	577.13513	−0.03	Procyanidin B2 ^abc^
18	C_15_H_14_O_6_	12.14	289.07176	289.07172	−0.14	Epicatechin ^abc^
19	C_21_H_22_O_11_	13.67	449.10893	449.10889	−0.09	Astilbin ^a^
20	C_45_H_38_O_18_	22.33	865.19854	865.19854	0.00	Procyanidin trimer C1 ^ab^
21	C_21_H_20_O_12_	25.39	463.0882	463.0882	0.00	Hyperoside ^ab^
22	C_27_H_30_O_16_	25.42	609.14611	609.14618	0.11	Rutin ^ab^
23	C_21_H_20_O_12_	25.79	463.0882	463.0882	0.00	Quercetin 3-O-glucoside ^abc^
24	C_15_H_10_O_6_	26.27	285.04046	285.04135	3.12	Kaempferol ^ab^
25	C_16_H_14_O_4_	27.81	269.08193	269.08218	0.93	Pinostrobin ^a^
26	C_15_H_10_O_5_	28.01	269.04555	269.04523	−1.19	Apigenin ^ab^
27	C_21_H_20_O_11_	28.3	447.09328	447.09323	−0.11	Quercetin 3-O-rhamnoside ^abc^
28	C_28_H_34_O_15_	29.93	609.18249	609.1825	0.02	Hesperidin ^a^
29	C_12_H_16_O_7_	47.50	271.08233	271.08295	2.29	Arbutin ^a^

^a^ compared with database; ^b^ compared with literatures; ^c^ compared with authentic standards.

**Table 4:** Contents of major phenolic compounds in different kiwifruit extracts.

Compounds	Regression Equation	R^2^	Linear Range (μg/mL)	Retention Time (min)	UDE (mg/g DW)	UEE (mg/g DW)	EE (mg/g DW)
**GA**	y = 72.203x + 645.05	0.9962	2.4–38.5	8.32	0.951 ± 0.042 ^a^	0.287 ± 0.019 ^b^	0.108 ± 0.034 ^c^
**PA**	y = 27.56x + 604.29	0.9965	4.9–76.9	16.55	1.226 ± 0.022 ^a^	0.384 ± 0.053 ^b^	0.026 ± 0.037 ^c^
**PB1**	y = 1.6993x + 49.478	0.9984	4.9–76.9	21.63	1.947 ± 0.134 ^a^	0.855 ± 0.062 ^b^	0.276 ± 0.012 ^c^
**Ca**	y = 7.0168x + 48.739	0.9994	4.9–76.9	24.93	0.914 ± 0.102 ^a^	0.259 ± 0.079 ^b^	0.086 ± 0.011^c^
**PB2**	y = 6.4073x + 350.22	0.9971	4.9–76.9	28.76	4.799 ± 0.128 ^a^	1.635 ± 0.081 ^b^	0.644 ± 0.057 ^c^
**EC**	y = 10.786x + 121.29	0.9978	4.9–76.9	30.4	1.583 ± 0.164 ^a^	1.264 ± 0.139 ^b^	0.470 ± 0.063 ^c^
**NCHL**	y = 45.262x + 970.83	0.9969	4.9–76.9	34.09	1.512 ± 0.112 ^a^	0.732 ± 0.097 ^b^	0.280 ± 0.047 ^c^
**CHL**	y = 35.003x + 425.98	0.9988	4.9–76.9	36.76	0.732 ± 0.093 ^a^	0.207 ± 0.062 ^b^	0.069 ± 0.013 ^c^
**QGlu**	y = 23.737x + 319.63	0.9962	4.9–76.9	54.12	0.708 ± 0.064 ^a^	0.222 ± 0.034 ^b^	0.074 ± 0.018 ^c^
**QRha**	y = 18.549x + 227.08	0.9961	4.9–76.9	57.45	0.696 ± 0.051 ^a^	0.271 ± 0.077 ^b^	0.088 ± 0.024 ^c^
**Total content (mg/g DW)**					15.067 ± 1.143 ^a^	6.122 ± 0.074 ^b^	2.218 ± 0.271 ^c^

GA, gallic acid; PA, protocatechuic acid; PB1, procyanidin B1; Ca, catechin; PB2, procyanidin B2; EC, epicatechin; NCHL, neochlorogenic acid; CHL, chlorogenic acid; QRha, Quercetin-3-rhamnoside; QGlu, quercetin-3-O-glucoside; UDE, the thinned kiwifruit extract prepared by ultrasound-assisted deep eutectic solvent extraction; UEE, the thinned kiwifruit extract prepared by ultrasound-assisted ethanol extraction; EE, the thinned kiwifruit extract prepared by conventional organic solvent extraction; different letters (a–c) in the same column indicate significant differences at *p* < 0.05 determined by ANOVA.

## Data Availability

All of the Data is contained within the article.

## Abbreviations

ABTS: 2,2′-azino-bis (3-ethylbenzothiazoline-6-sulphonic acid); ANOVA, analysis of variance; BBD, Box-Behnken design; Ca, catechin; CE/g DW, the catechin equivalent per gram of kiwifruit dry weight; ChCl, choline chloride; CHL, chlorogenic acid; CSE, conventional organic solvent extraction; DES, deep eutectic solvents; DMEM, Dulbecco’s modified eagle medium; DPPH, 2,2-diphenyl-1-picrylhydrazyl; DW, dry weight; EC, epicatechin; EE, the thinned kiwifruit extract prepared by conventional organic solvent extraction; FRAP, ferric-reducing antioxidant power; GA, gallic acid; GAE/g DW, the gallic acid equivalent per gram of kiwifruit dry weight; Gly, glycerol; IL-6, interleukin-6; LPS, lipopolysaccharide; MTT, 3-(4,5-dimethylthiazol-2-yl)-2,5-diphenyltetrazolium bromide; NCHL, neochlorogenic acid; NO, nitric oxide; OH, hydroxyl radical; PA, protocatechuic acid; PB1, procyanidin B1; PB2, procyanidin B2; PL, pancreatic lipase; QRha, quercetin-3-rhamnoside; QGlu, quercetin-3-O-glucoside; RE/g DW, the rutin equivalent per gram of kiwifruit dry weight; RSM, response surface methodology; TPC, total phenolic content; TFC, total flavonoid content; TPAC, total procyanidin content; TNF-α, tumor necrosis factor-alpha; UADE, ultrasound-assisted deep eutectic solvent extraction; UAEE, ultrasound-assisted ethanol extraction; UDE, the thinned kiwifruit extract prepared by ultrasound-assisted deep eutectic solvent extraction; UEE, the thinned kiwifruit extract prepared by ultrasound-assisted ethanol extraction; UPLC-MS/MS, ultra-performance liquid chromatography-tandem mass spectrometry.
